# Young adults with colon cancer: clinical features and surgical outcomes

**DOI:** 10.1186/s12876-023-02770-y

**Published:** 2023-06-03

**Authors:** Chao Wang, Lin Gan, Zhidong Gao, Zhanlong Shen, Kewei Jiang, Yingjiang Ye

**Affiliations:** Department of Gastrointestinal Surgery, Peking University People’s Hospital, 11 Xizhimen Nan Street, Xicheng District, Beijing, 100044 P. R. China

**Keywords:** Colon cancer, Young-onset patient, Surgery, Clinicopathologic characteristics, Survival

## Abstract

**Background:**

The clinicopathological features, surgical outcomes, and long-term survival of patients with young-onset colon cancer (≤ 40 years old) remain controversial.

**Methods:**

The clinicopathologic and follow-up data of patients aged < 40 years with colon cancer between January 2014 and January 2022 were reviewed. The primary objectives were clinical features and surgical outcomes. Long-term survival was investigated as a secondary objective.

**Results:**

Seventy patients were included in the study, and no significant rising trend (Z=0, *P*=1) of these patients was observed over the 8-year study period. Stage IV disease was accompanied by more ulcerative or infiltrating type (84.2% vs. 52.9%, *P*=0.017) and lymphovascular or perineural invasion (64.7% vs. 25.5%, *P*=0.003) than stage I–III disease. After a median follow-up time of 41 months (range 8–99 months), the 1-, 3-, and 5-year estimated overall survival (OS) rates were 92.6%, 79.5%, and 76.4%, respectively. The 1-, 3-, and 5-year progression-free survival (PFS) rates were 79.6%, 71.7%, and 71.7%, respectively. Multivariate Cox regression showed that M+ stage (hazard ratio [HR], 3.942; 95% confidence interval [CI], 1.176–13.220, *P*=0.026) was the only independent risk factor affecting OS. Meanwhile, tumor deposits (HR, 4.807; 95% CI, 1.942–15.488, *P*=0.009), poor differentiation (HR, 2.925; 95% CI, 1.012–8.454, *P*=0.047), and M+ stage (HR, 3.540; 95% CI, 1.118–11.202, *P*=0.032) independently affected PFS.

**Conclusions:**

The differences in the clinical features, surgical outcomes, and long-term survival between young adults and elderly colon cancer patients need further investigation.

## Background

Colon cancer is the fifth most common malignant disease, accounting for 6% of new cancer cases and 5.8% of new cancer deaths worldwide [1]. Many risk factors, such as age, race, accompanying inflammatory bowel disease, and a family history of gastrointestinal cancer, contribute to the incidence of colon cancer [2, 3]. Among them, increasing age is considered the major unchangeable risk factor for sporadic colon cancer; nearly 70% of patients are >65 years of age, and this disease is rare before the age of 40 years [4]. However, this trend has changed, and the incidence and death rates of colorectal cancer in younger individuals have been rising [5]. A population-based study [6] from England showed a six-fold increase in younger colorectal cancer patients over the past three decades, and the most sustained increase in the incidence rate was in the group aged 20–29 years. The presentation, tumor biology, and survival pattern of young-onset patients were reported to be different from those of the older population [7, 8], which might bring challenges to oncologists because of the more advanced stage, more tolerance to therapy, and longer life expectancy of young-onset patients. Previous studies [9, 10] on this topic were mainly based on public databases from Western countries and focused on the description of epidemiological or demographic data, lacking the analysis of clinicopathological features, treatment strategy, and surgical outcomes, especially in advanced tumors among Asian patients.

Given the deficiency of previous studies, here we aimed to identify the clinicopathological features and long-term survival in young-onset colon cancer patients (≤ 40 years old), which would be beneficial for providing abundant evidence for precision treatment.

## Materials and methods

### Study design and participants

This retrospective study was conducted following the STROBE statement [11] and was approved by the Ethics Committee of Peking University People’s Hospital (Beijing, China). The medical records of patients with colon cancer treated at Peking University People’s Hospital between January 2014 and January 2022 were reviewed.

The inclusion criteria were age ≤ 40 years and primary colon cancer. Exclusion criteria were hereditary colorectal cancer syndrome, inflammatory bowel disease, and other malignant diseases.

### Process of patient’s management and follow-up

All patients were diagnosed and preoperatively staged using colonoscopic biopsy and contrast-enhanced computed tomography (CT; chest, abdomen, and pelvic). Positron emission tomography-CT was used for patients with metastatic disease, as appropriate. Patient management was conducted under the advice of a multidisciplinary team.

The final follow-up for all patients was performed in June 2022 via telephone or recent laboratory tests to evaluate survival and oncological status. Overall survival (OS) is the time from treatment until death, and progression-free survival (PFS) is the time from treatment until investigator-assessed radiological disease progression.

### Statistical analysis

Continuous variables are presented as medians (first to third quartiles), and categorical variables are presented as frequencies with percentages. Continuous and categorical variables were performed using the Mann–Whitney *U* test and Pearson’s chi-squared test between two groups, respectively.

The Mann–Kendall test was used to test whether the change in the included cases had a significant trend over the years. OS and PFS curves were created, and 1-, 3-, and 5-year OS and PFS rates were calculated. Univariate and multivariate Cox regression models were used to assess the OS and PFS risk factors, and variables with *P*<0.05 in univariate analyses were included in multivariate analyses.

All statistical analyses were conducted using the R software (version 4.1.2; R Foundation for Statistics Computing, Vienna, Austria). A two-sided *P*<0.05 was considered statistically significant. There were no missing data in this study.

## Results

### Patients’ enrollment and baseline characteristics

**Fig. 1:**
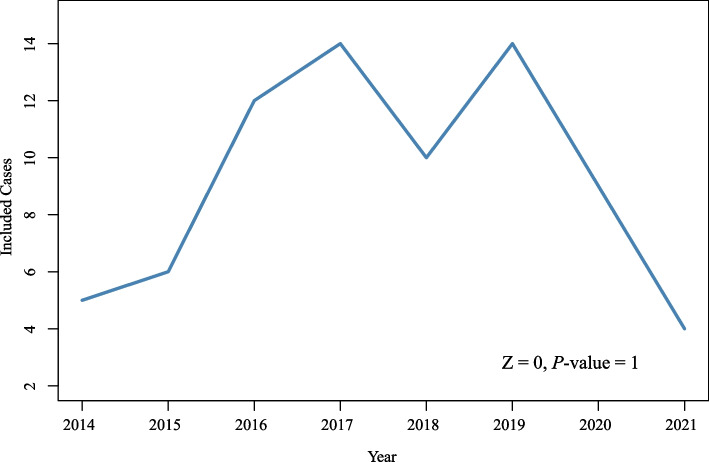
Line plot of included cases per year

Seventy patients met the inclusion and exclusion criteria for this study, and no significant rising trend (Z=0, *P*=1) in the number of young-onset colon cancer patients was observed over 8 years (Fig. 1). The baseline characteristics of the patients are shown in Table 1. Stage IV disease was accompanied by a more ulcerative or infiltrating type (84.2% vs. 52.9%, *P*=0.017) and lymphovascular or perineural invasion (64.7% vs. 25.5%, *P*=0.003) than stage I–III diseases. In contrast, patients with stage I–III disease had more MMR-deficient (31.4% vs. 5.9%, *P*=0.036) and a higher proportion of receiving surgery as the initial treatment (100.0% vs. 52.6%, *P*<0.001).

**Table 1:** Baseline and pathological characteristics

	Stage I-III colon cancer (*n* = 51)	Stage IV colon cancer (*n *= 19)	*P* value
Age, years	36 (32-38)	36 (33-38)	0.745
Male	25 (49.0)	10 (52.6)	0.788
Tumor history	16 (31.4)	6 (31.6)	0987
Right-sided CC	21 (41.2)	9 (47.4)	0.642
Tumor diameter, cm	5 (3-7)	5 (3-8)	0.865
Ulcerative or infiltrating type	27 (52.9)	16 (84.2)	**0.017**
Mucinous adenocarcinoma	11 (21.6)	5 (25.3)	0.674
Poor differentiation	14 (27.5)	8 (42.1)	0.240
Tumor deposits^a^	8 (15.7)	4 (23.5)	0.463
Lymphovascular or perineural invasion^a^	13 (25.5)	11 (64.7)	**0.003**
MMR-deficient^a^	16 (31.4)	1 (5.9)	**0.036**
Surgery as the initial treatment	51 (100.0)	10 (52.6)	**< 0.001**

Data are presented as median (first to third quartile) and numbers (%)

*P*-values for age and tumour diameter were calculated using the Mann–Whitney *U* test; other *P*-values were calculated using Pearson’s chi-squared test

^a^Two stage IV colon cancer patients did not have colectomy during treatment

### Treatment characteristics

As shown in Table 2, 37.3% (19/51) of tumors penetrated the visceral peritoneal layer or adjacent organs, and 39.2% (20/51) of the tumors had lymph node metastasis. All patients underwent complete mesocolic excision, and two of them had postoperative complications. One patient was a 30-year-old male with ascending colon cancer, who had incision fat liquefaction on the sixth day after surgery and was cured after wound dressing. The other patient was a 26-year-old female with sigmoid colon cancer who developed gastroparesis after surgery and experienced 27 days of postoperative hospital stay for conservative treatment. The number of colon cancer patients that received adjuvant chemotherapy was 17/20 (stage III) and 6/10 high-risk stage II.

**Table 2:** Treatment and follow-up of stage I-III colon cancer

	Stage I-III colon cancer (*n* = 51)
T stage	
1	11 (21.6)
2	5 (9.8)
3	16 (31.3)
4a	14 (27.5)
4b	5 (9.8)
Local invasion	5 (9.8)
Abdominal muscle	1 (2.0)
Ureter	2 (3.9)
Ileum	2 (3.9)
N stage	
0	31 (60.8)
1	13 (25.5)
2	7 (13.7)
UICC stage	
I	13 (25.5)
II	18 (32.3)
III	20 (39.2)
Total LN harvest	25 (15-45)
Postoperative surgical complications	2 (3.9)
Wound complications	1 (2.0)
Gastroparesis	1 (2.0)
Length of postoperative hospital stay, d	8 (7-10)
Adjuvant chemotherapy^a^	23 (45.1)
Follow-up time, months	44 (30-67)
Death	6 (11.8)
Disease progress	7 (13.7)

Data are presented as median (first to third quartile) and numbers (%)

*Abbreviations*: *LN* Lymph node

^a^3 stage III and 4 high-risk stage II colon cancer patients did not accept adjuvant chemotherapy, respectively

As shown in Table 3, the most frequent metastatic pattern was peritoneum plus one or more organs (47.7%, 9/19). Nine patients underwent conversion therapy, and the others underwent surgery first. Simple colectomy was performed in 5 (26.3%) patients, and 12 (63.2%) patients underwent multi-visceral resection. Four patients had postoperative surgical complications, two had intraperitoneal infections, and one had wound complications. Two patients had peritoneal metastases and underwent cytoreductive surgery with hyperthermic intraperitoneal chemotherapy. The last surgical complication was urine leakage, which occurred in a 36-year-old patient with sigmoid colon cancer that invaded the bladder.

**Table 3:** Treatment and follow-up of stage IV colon cancer

	Stage IV colon cancer (*n* = 19)
Metastatic organ at initially diagnosed	
Liver	6 (31.6)
Retroperitoneal LN	1 (5.3)
Peritoneum	3 (15.7)
Peritoneum and other organ(s)	9 (47.4)
Surgical procedure	
Colectomy	5 (26.3)
Colectomy + CRS ± HIPEC	7 (36.9)
Colectomy + partial hepatectomy	5 (26.3)
Colostomy	2 (10.5)
T stage	
3	5 (26.2)
4a	7 (36.9)
4b	7 (36.9)
Local invasion	
Abdominal muscle	2 (10.5)
Ileum	1 (5.3)
Pancreas	1 (5.3)
Ureter	1 (5.3)
Bladder	3 (15.6)
N stage	
0	4 (21.1)
1	8 (42.1)
2	5 (26.3)
Total LN harvest	33 (23-52)
Postoperative surgical complications	4 (21.1)
Intraperitoneal infection	2 (10.5)
Wound complication	1 (5.3)
Urine leakage	1 (5.3)
Length of postoperative hospital stay, d	11 (8-16)
Follow-up time, months	22 (10-54)
Death	8 (42.1)
Disease progress	12 (63.2)

Data are presented as median (first to third quartile) and numbers (%)

*Abbreviations*: *CRS* Cytoreductive surgery, *HIPEC* Hyperthermic intraperitoneal chemotherapy

### Survival analysis

**Fig. 2:**
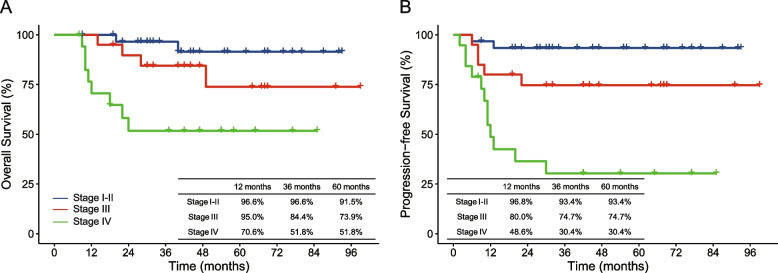
Survival curves of patients stratified for disease stage. Tables show the estimated survival rate at 12, 36, and 60 months. **A** Overall survival curve, **B** Progression-free survival curve

All patients had a median follow-up period of 41 months (range 8–99 months). For stage I–III disease, six and seven patients experienced death and disease progression, respectively. Of the patients who died, two died of leukemia, and the others died of colon cancer. As for stage IV disease, 8 and 12 patients experienced death and disease progression, respectively. All the stage IV disease deaths were from colon cancer. The 1-, 3-, and 5-year OS rates of all patients were 92.6%, 79.5%, and 76.4%, respectively. The 1-, 3-, and 5-year PFS rates of all patients were 79.6%, 71.7%, and 71.7%, respectively. Figure 2 shows the 1-, 3-, and 5-year OS and PFS rates stratified by stage. Table 4 shows the univariate Cox regression model analyses of the OS and PFS. In multivariate Cox regression analyses, M+ stage (hazard ratio [HR], 3.942; 95% confidence interval [CI], 1.176–13.220; *P*=0.026) was the only independent risk factor for OS. Meanwhile, tumor deposits (HR, 4.807; 95% CI, 1.942–15.488, *P*=0.009), poor differentiation (HR, 2.925; 95% CI, 1.012–8.454, *P*=0.047), and M+ stage (HR, 3.540; 95% CI, 1.118–11.202, *P*=0.032) independently affected PFS.

**Table 4:** Univariate cox regression model analyses of OS and PFS

	HR (95% CI)	*P*-value
Overall survival		
Poor differentiation	2.913 (1.008-8.421)	0.048
N+ stage	3.839 (1.070-13.768)	0.039
M+ stage	5.428 (1.876-15.706)	0.002
Progression-free survival		
Ulcerative or infiltrating type	3.696 (1.076-12.689)	0.038
Poor differentiation	3.060 (1.240-7.551)	0.015
Tumor deposits	4.345 (1.650-11.439)	0.003
Lymphovascular or perineural invasion	3.761 (1.389-10.180)	0.009
N+ stage	4.187 (1.389-12.621)	0.011
M+ stage	6.913 (2.697-17.721)	<0.001

*Abbreviations*: *OS* Overall survival, *PFS* Progression-free survival, *HR* Hazard ratio, *CI* Confidence interval

## Discussion

Recent studies [12, 13] have emphasized an increased number of patients diagnosed with colon cancer in their 20s, 30s, and 40s. In the past, tumor biology and survival patterns were different between young colon cancer adults and the older population, which was mainly due to the more aggressive tumor biology. This would result in more intensive treatment measures because of greater tolerance to therapy and a stronger will to survive compared with older patients. However, a few retrospective studies [10, 12, 14] showed that young colon cancer patients tend to have similar surgical outcomes and OS compared to older patients. Therefore, investigations of surgical outcomes and long-term survival in young patients with colon cancer are necessary to provide abundant evidence for precision treatment. Our findings show that colon cancer in young adults is not unique. Differences in clinical features, surgical outcomes, and long-term survival were not observed in this population.

The incidence of young-onset colon cancer in adults with varies across countries and regions. A population-based study [9] that reviewed data in England from 2010 to 2014 found that among 167501 colorectal cancer patients, 3657 (2.2%) patients were 40 years old or younger, with an increasing trend in the proportion of young-onset patients (14.4% in 2010 to 23.7% in 2014, *P*<0.05). Another study [12] analyzed data from the Mayo Clinic Cancer Registry from 1972 to 2017 and found that the percentage of patients aged < 50 years diagnosed with rectal cancer increased linearly at a rate of 0.26% per year (*P*<0.001); however, a similar trend was not observed in colon cancer (*P*=0.296). In contrast to the Mayo Clinic, data from the Chinese Cancer Registry Annual Report showed consistent trends of average annual percent change from 2005 to 2015 in colon (0.9, 95% CI -0.6 to 2.4) and rectal (-0.5, 95% CI -2.1 to 1.1) cancer patients aged 20–34 [15]. In the present study, we also found no significant increasing trend (*P*=1) in young-onset colon cancer patients. Although the incidence of young-onset colon cancer is controversial, further studies should be performed to explore the potential influencing factors associated with the epidemiology of younger individuals at greater risk, and the current screening recommendations should also be reconsidered.

Conventional viewpoints treat young-onset colon cancer as a particular type with low incidence, which is always accompanied by extremely aggressive tumor biology and poor prognosis. However, a recent study [9] with the largest sample of young-onset colorectal cancer patients showed that only 16.9% of cases were poorly differentiated, and 28.7% of cases had metastatic disease. Another study [10] investigated 947 extremely young (≤25 years) colon cancer patients by reviewing the National Cancer Database and found that 29.7% of patients had poor tumor histology (mucinous, mucin-producing, or signet ring cell adenocarcinoma), and 27.5% of patients had metastatic disease. Our study had similar results, with 31.4% showing poor differentiation, 22.5% mucinous adenocarcinoma, and 27.1% metastatic disease. Thus, preconceptions about extremely aggressive tumor biology accompanied by young-onset colon cancer should be abandoned; colon cancer occurring in young adults was rare but not a particular type compared to the older ones.

Surgery is the primary treatment for non-metastatic colon cancer, and no unique surgical procedure has been proposed for patients with young-onset colon cancer. In the present study, all patients with stage I–III disease underwent complete mesocolic excision as the initial treatment. The short-term surgical outcomes, including a 3.9% surgical complication rate and 8 days of postoperative hospital stay, were acceptable and similar to previously published studies from our center [16] or others [17, 18] that performed complete mesocolic excision for all age groups. Satisfied long-term survival for early stage young-onset colon cancer patients was revealed by a recent study [9] with a large sample size, which showed 98.2% and 89.1% 5-year overall survival for stage I and II disease, respectively. In the present study, stage I and II patients also had satisfactory prognoses, with an estimated 91.5% 5-year OS and 93.5% 5-year PFS. As for stage III disease, a large sample size study [10] from the USA showed a 60.6% 5-year OS, which was inferior compared to our study of 73.9% 5-year OS and the result of 74.8% from England [9]. This might be because the USA study only included patients aged ≤ 25 years, which was much younger than our study and the England study. Second, as illustrated in the USA study, it contained a higher proportion of Black patients, and young Black patients have also been found to have worse overall survival outcomes compared to non-Hispanic Whites [10, 19]. An English study also showed that ethnicity affected the 5-year OS, with Black 63.0%, Caucasians 71.2%, and Chinese 76.2% (*P*<0.001).

The treatments for stage IV disease in young-onset colon cancer may vary from those in older patients in clinical practice. In most regions, more intensive treatment is applied for young adults because of greater tolerance to therapy and longer life expectancy [20]. However, a study [14] reanalyzed the Cancer and Leukemia Group B and SWOG 80405 trial and found there were no significant differences between colorectal cancer patients aged ≤ 50 years and >50 years regarding median OS (27.07 vs 26.12 months, *P*=0.78) and PFS (10.87 vs 10.55 months, *P*=0.67) during a median follow-up time of 5.98 years. In the present study, nine patients received surgery and others received conversion therapy as the initial treatment, and none received palliative treatment. The estimated 5-year OS of 51.8% was also longer than that of the England [9] (20.1%) and USA [10] (14.1%) studies. The most likely reason might be that patients with stage IV disease whose PFS was longer than 60 months were all isolated liver metastases and underwent radical surgery for primary and metastatic lesions simultaneously. Therefore, radical surgery may be the first choice for patients with colon cancer with isolated liver metastasis.

This study had some limitations. First, limited by the study design, we could not collect the data of older patients to make a comparison, which might make the conclusion less reliable. Second, the retrospective nature and small sample size could have caused bias and impacted external adaptation. Third, our study lacks the analysis of the influence of molecular mechanisms or pathway mutations in young-onset colon cancer patients which might illustrate the pathogenesis and could be the final indication of the difference between young-onset and older colon cancer. Last, the limited sample size could result in potential bias, which only large nationwide databases could address this important issue.

In summary, The differences in the clinical features, surgical outcomes, and long-term survival between young adults and elderly colon cancer patients need further investigation.

## Data Availability

The original data of the published results can be shared if needed by other researchers and can be requested by emailing the corresponding author on yeyingjiang@pkuph.edu.cn.

## Abbreviations

CI: Confidence interval
CT: Computed tomography
HR: Hazard ratio
OS: Overall survival
PFS: Progression-free survival
